# Continued growth after fixation of slipped capital femoral epiphysis

**DOI:** 10.1007/s11832-016-0793-x

**Published:** 2016-11-05

**Authors:** Per Holmdahl, Torsten Backteman, Aina Danielsson, Johan Kärrholm, Jacques Riad

**Affiliations:** 1 Department of Orthopaedics, Skaraborg Hospital, 541 85 Skövde, Sweden; 2 Institute of Clinical Sciences, Sahlgrenska Academy, University of Gothenburg, Gothenburg, Sweden; 3 Department of Paediatric Surgery, The Queen Silvia Children’s Hospital, Sahlgrenska University Hospital, Gothenburg, Sweden; 4 Department of Orthopaedics, Sahlgrenska University Hospital, Gothenburg, Sweden

**Keywords:** Slipped capital femoral epiphysis, Growth, Remodelling, Impingement, Osteoarthritis, Radiostereometric analysis

## Abstract

**Purpose:**

When treating slipped capital femoral epiphysis (SCFE), a smooth pin with a hook or a short threaded screw can be used to allow further growth, which could be important to prevent the development of impingement and early arthritis. The purpose of this investigation was to measure growth in three dimensions after fixation of SCFE.

**Methods:**

Sixteen participants with unilateral SCFE, nine girls and seven boys with a median age of 12.0 years (range 8.4–15.7 years), were included. The slipped hip was fixed with a smooth pin with a hook, and the non-slipped hip was prophylactically pinned. At the time of surgery, tantalum markers were installed bilaterally on each side of the growth plate through the drilled hole for the pin. Examination with radiostereometric analysis (RSA) was performed postoperatively and at 3, 6 and 12 months. The position of the epiphysis in relation to the metaphysis was calculated.

**Results:**

At 12 months, the epiphysis moved caudally, median 0.16 mm and posteriorly 2.28 mm on the slipped side, in comparison to 2.28 cranially and 0.91 mm posteriorly on the non-slipped side, *p* = 0.003 and *p* = 0.030, respectively. Both slipped and non-slipped epiphysis moved medially, 1.52 and 1.74 mm, respectively. A marked variation in the movement was noted, especially on the slipped side.

**Conclusions:**

The epiphysis moved in relation to the metaphysis after smooth pin fixation, both on the slipped side and on the prophylactically fixed non-slipped side, implying further growth. The RSA method can be used to understand remodelling after ‘growth-sparing’ fixation of SCFE.

## Background

The long-term goal in the treatment of slipped capital femoral epiphysis (SCFE) is to prevent the development of deformity with the risk of impingement, leading to early osteoarthritis [1–4]. Nevertheless, a screw is often used and, in the majority of cases, applied deliberately with compression over the growth plate to achieve closure [5–7]. Different fixation principles can be used which allows for further growth. In the Scandinavian countries, fixation is achieved with a smooth Hansson pin, which is supplied with a hook device to gain purchase in the epiphysis [8]. In addition, Kumm et al. describe a dynamic short thread screw fixation with only threads in the epiphysis, without compression over the growth plate to allow for potential growth [9].

Continued growth after fixation for SCFE was first described by Key [10]. Hägglund et al. measured longitudinal growth of the femoral neck and reported up to 15 mm of growth after pinning with the smooth pin [11]. The development of leg length discrepancy could be avoided and remodelling may have a positive effect on the long-term outcome [12–15].

The extent of remodelling has been investigated using various techniques [16, 17]. The most common method is conventional radiography and measurement of the head-to-shaft angle (HSA) [18]. These methods are, however, associated with comparatively large measurement errors [19]. Radiostereometric analysis (RSA) measures skeletal movements with high resolution. This method is based on the use of fixed skeletal markers (tantalum spheres, ø = 0.8 mm) and simultaneous radiographic exposure of the hip with the use of two X-ray tubes and a calibration system [20].

We consider that a fixation for SCFE that prevents further slippage, while allowing for continued growth with possible remodelling and prevention of future impingement, is ideal. It is, therefore, important to determine both the amount of growth and in which direction this occurs, something which is possible using the RSA technique.

The aim of this study was to use the RSA method to measure continued growth after fixation of SCFE with the smooth pin on both the slipped side and on the opposite, prophylactically fixed, non-slipped side.

The research questions were, accordingly:

1. Can RSA be used to measure growth in three dimensions after fixation of SCFE?
2. How much will the position of the epiphysis change over time in three dimensions compared to the postoperative position?
3. Can any differences be found between the slipped side and the non-slipped side?

## Method

### Procedure

Patients presenting with unilateral SCFE at the hospitals in Skaraborg (*n* = 7) and Borås (*n* = 1), as well as in Gothenburg (*n* = 8), Sweden, were recruited. Inclusion criteria were unilateral SCFE. Exclusion criteria were severe mental retardation and inability to understand the Swedish language. Ethical approval was obtained from the National Ethical Committee. Patients were included after receiving oral and written information and obtaining signed parental consent and the consent of the children whenever they were able.

### Patients

Of the 16 participants included, nine were girls and seven boys, with a median age of 12.0 years (range 8.4–15.7 years) (Table 1).

**Table 1:** Demographics of the patients

Age at surgery (years)	Gender	Weight (kg)	Stable	Degree of slip (°)
11.1	Female	Unknown	1	30
14.6	Male	Unknown	1	88
12.9	Female	41	1	59
11.7	Male	50	1	20
11.1	Female	50	0	36
13.4	Female	38	1	25
12.1	Female	53	1	51
15.7	Male	76	1	31
12.1	Male	66	0	38
15.1	Male	84	0	75
11.6	Male	34	1	14
10.7	Male	38	1	87
11.1	Female	54	1	15
12.0	Female	52	1	26
12.8	Female	56	1	38
8.4	Female	55	1	21

Preoperative radiographs and postoperative magnetic resonance imaging (MRI) confirmed unilateral involvement. Conventional radiographs revealed the slip and MRI was used to rule out oedema on the non-slipped side. Oedema would be interpreted as possible involvement of SCFE, even though no slip was noted [21]. Four hips were regarded as unstable and these patients used a crutch to avoid weight-bearing. The degree of slippage was measured on the lateral radiographs according to Southwick [18]. The median slip was 34° (range 14°–88°) (Table 1).

### Surgical procedure

**Fig. 1:**
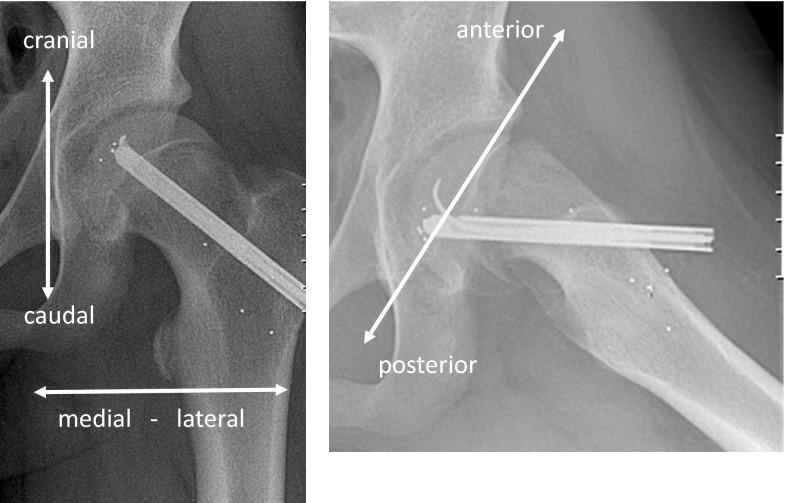
Postoperative frontal and sagittal view, with tantalum markers inserted as well as the smooth hook pin (Hansson) on the slipped side

Patients had surgical treatment within two days from diagnosis. Hansson pinning was performed by one of three orthopaedic surgeons, all with a long experience of the smooth pin system. Prophylactic pinning of the non-slipped side was performed on all patients. During surgery, four to nine spherical tantalum markers (ø = 1.0 mm) were inserted on each side of the growth plate on both the slipped and non-slipped sides. These markers were installed through the drilled canal (6.5 mm diameter) for the smooth pin. A specially designed long nozzle attached to an RSA pistol (RSA Biomedical, Umeå, Sweden) was used to insert the tantalum markers into the epiphyseal and metaphyseal regions (Fig. 1). The first RSA examination was performed within one week after surgery, and thereafter at 3 6 and 12 months, and then annually until skeletal maturity. Patients were allowed to bear weight after surgery but were not permitted to participate in sports during the first 12 weeks.

### Radiostereometric analysis (RSA)

The patients were examined in a radiographic laboratory primarily designed for RSA (Adora RSA, NRT-Nordisk Røntgen Teknik, Hasselager, Denmark) with use of digital screens (Canon CXDI-50RF, 5.9 pixels, 4096 greyscale, 12-bit). We used the uniplanar technique (cage 77, UmRSA Biomedical, Umeå, Sweden). Exposures were 133 kV and 5 mA.

Two X-ray tubes angled at about 35° in relation to each other were used to examine the hips with the patient supine. A calibration cage was placed between the examination table and the digital screens. Tantalum markers in this cage defined the laboratory coordinate system. These markers with known positions were also used to compute the position of the X-ray foci. This information and the two-dimensional position of the patient markers on the digital images could be used to calculate the three-dimensional coordinates of each patient marker at each examination. If the bone markers were found to maintain their positions in relation to each other between examinations in one and the same hip (i.e. markers were found to be stable), the absolute motions (rotations and translations) of the metaphyseal markers were used to ‘replace’ the bone at the follow-up studies to its original position present at the first examination by the use of an inverse rotation matrix. After performing this mathematical computation resulting in a closed to unchanged position of the proximal femoral metaphysis throughout the entire follow-up, the relative femoral motions were calculated.

The resolution of RSA examinations are dependent on several factors, e.g. image quality, marker stability and marker scatter. Sufficient marker scatter is of particular importance for the evaluation of rotations. (It is easy to realise that the closer the markers are located along a straight line, the more difficult it will be to determine rotations along this line.) Marker scatter is evaluated based on a mathematical formula which computes a so-called condition number. The smaller this number, the better the scatter. In the present study, we regarded the marker scatter too poor in the bone segment chosen to be moving (the epiphysis). We thought that computation of rotations not could be done with reasonable resolution in the majority of hips. Therefore, we only present translations, which only require well-scattered markers in the reference segment (the metaphysis) and marker stability in both segments. Marker stability is calculated by determination of the mean error of rigid body fitting [20, 22]. Details for those who use the RSA method are presented below.

In the metaphyseal region, the marker scatter corresponded to a condition number of median 72. In the epiphyseal region, the marker scatter was lower since the markers were inserted at the end of the drill hole, through the femoral neck, into the epiphyseal segment, with a condition number of median 185. We did not want to risk any damage to the growth plate or cartilage by the insertion of markers in any other way. Because of lower marker scatter in the epiphysis, we decided to restrict the evaluation to translations, not including rotation. A median of 6 (3–9) markers could be evaluated in the reference (metaphysis) and 5 (3–8) in the moving segment (epiphysis). The maximum accepted mean errors of the reference and moving segments was set at 0.350 mm and the observed median values were 0.177 (0.018–0.344) and 0.105 (0.010–0.330).

In this way, we take into account medial (+)/lateral (−), cranial (+)/caudal (−) and anterior (+)/posterior (−) motions of the gravitational centre of tantalum markers located close to the tip of the pin.

The precision of RSA measurements is calculated based on repeated examinations of the hip at the same occasion. The patient should be repositioned between the examinations. If the epiphysis is stable between the examinations which are performed with an interval of up to 15 min, the motions recorded between these examinations should be zero. Due to, for example, differences in film quality, measurement errors and other factors, there will always be a small difference. The precision of translation measurements in this study was based on repeated examinations (double examinations) in 28 hips. The 99% detection limits in the individual case [2.8 times the standard deviation (SD) of the error] around a mean value of zero, were 0.21, 0.24 and 0.65 mm in the medial/lateral, proximal/distal and anterior/posterior directions, respectively. This means that, in the evaluation of individual cases, the recorded motions were true with 99% probability if exceeding these limits.

In this study, we report from the first 12 months of follow-up. The position of the epiphysis in relation to the metaphysis after 3 and 6 months and after 1 year was compared with the position immediately postoperatively. The movement was calculated for each individual and the median and range for the entire group on both the slipped and non-slipped sides. In addition, both the translation vector in the frontal plane (medial/lateral and cranial/caudal) and the vector in all three directions was calculated.

### Statistical methods

Results regarding continuous variables, as well as changes in continuous variables, are described with the median and range for each side, slipped and non-slipped. For the comparison of continuous variables between sides, the Wilcoxon signed-rank test was used. Correlations were analysed using the Spearman rank correlation. All tests were two-tailed and conducted at the 5% significance level. The data were analysed using SPSS Statistics 22. The study was approved by the Human Research Ethics Committee of the Medical Faculty at the University of Gothenburg, Sweden (778-10).

## Results

**Fig. 2:**
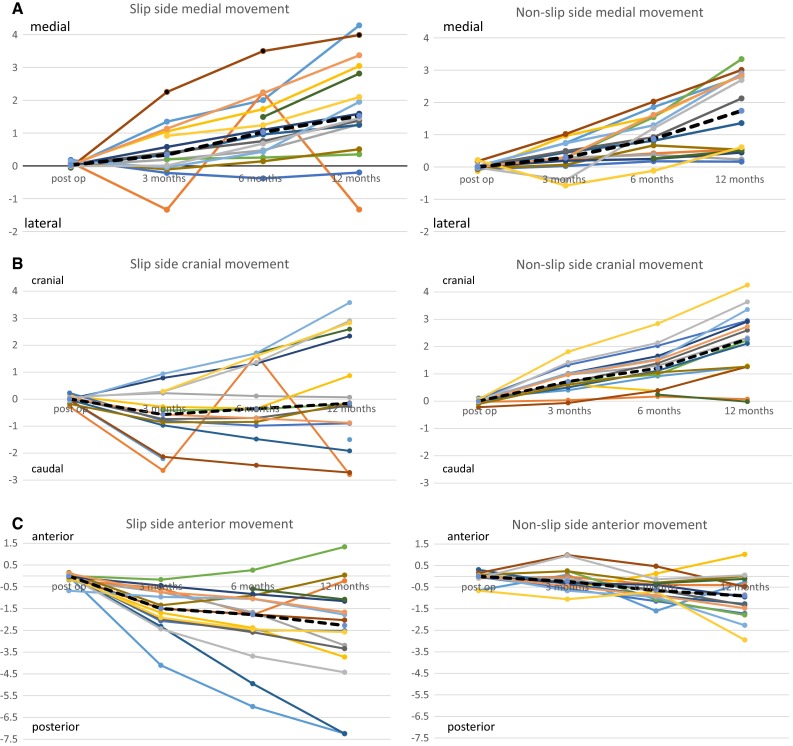
**a** Movement (in mm) on the slipped and non-slipped sides for each individual and the median (*black dotted line*) in the medial/lateral direction. *n* = 16. **b** Movement (in mm) on the slipped and non-slipped sides for each individual and the median (*black dotted line*) in the cranial/caudal direction. *n* = 16. **c** Movement (in mm) on the slipped and non-slipped sides for each individual and the median (*black dotted line*) in the anterior/posterior direction. *n* = 16

In 14 (out of 16) of the slipped hips, the epiphysis moved in the medial direction and two in the lateral direction, while on the non-slipped side, all 16 moved medially. The median movement was 1.52 mm medial (range 1.33 lateral to 4.28 medial) on the slipped side and 1.74 mm (range 0.16 medial to medial 3.34) on the non-slipped side, *p* = 0.717 (Tables 2 and 3; Fig. 2a).

**Table 2:** Movements for each patient and the median in three separate planes, in the two-plane (frontal) vector and in the three-plane vector, after 12 months on the slipped side and the non-slipped side

Slipped side					Non-slipped side				
Medial/lateral	Cranial/caudal	Anterior/posterior	Two-plane (frontal) vector	Three-plane vector	Medial/lateral	Cranial/caudal	Anterior/posterior	Two-plane (frontal) vector	Three-plane vector
4.28	−1.50	−7.24	4.53	8.54	2.81	1.26	−0.25	3.07	3.08
−1.33	−2.80	−0.23	3.10	3.11	0.55	0.07	−0.41	0.55	0.69
1.27	0.07	−3.19	1.27	3.44	0.23	2.32	−0.86	2.33	2.48
3.04	0.87	−3.73	3.16	4.89	2.86	1.29	1.02	3.14	3.30
−0.20	−0.90	−2.52	0.92	2.69	0.16	2.95	−1.73	2.95	3.42
0.35	−0.17	1.33	0.39	1.39	3.34	2.23	−1.79	4.02	4.40
1.25	−1.92	−7.25	2.29	7.60	1.36	2.11	−1.28	2.51	2.82
3.99	−2.72	−2.03	4.82	5.24	3.01	1.26	−0.48	3.26	3.29
1.38	−0.24	−3.34	1.40	3.62	2.12	2.60	−1.33	3.36	3.61
0.51	−0.14	0.03	0.52	0.52	0.53	1.27	0.01	1.37	1.37
1.59	2.34	−1.16	2.82	3.05	0.45	2.91	−0.95	2.95	3.10
2.81	2.60	−1.08	3.83	3.98	0.51	−0.02	−0.12	0.51	0.53
1.95	3.58	−1.78	4.07	4.45	2.90	3.36	−2.27	4.43	4.98
3.37	−0.88	−1.67	3.48	3.86	2.83	2.73	−1.48	3.93	4.20
1.46	2.90	−4.43	3.25	5.49	2.69	3.64	0.05	4.52	4.52
2.10	2.83	−2.58	3.52	4.36	0.61	4.25	−2.95	4.30	5.21

Medial direction = positive and lateral direction = negative

Cranial direction = positive and caudal direction = negative

Anterior direction = positive and posterior direction = negative

**Table 3:** Movement in the three different planes separately, in the two-plane (frontal) vector (vector of medial/lateral and cranial/caudal movement) and in the three-plane (total) vector, 12 months postoperatively

Direction	Slipped side		Non-slipped side		*p*-Value
	Median	Range	Median	Range	
Medial/lateral	1.52 medial	1.33 lateral to 4.28 medial	1.74 medial	0.16–3.34 medial	0.717
Cranial/caudal	0.16 caudal	2.80 caudal to 3.58 cranial	2.28 cranial	0.02 caudal to 4.25 cranial	0.003
Anterior/posterior	2.28 posterior	7.25 posterior to 1.33 anterior	0.91 posterior	2.95 posterior to 1.02 anterior	0.030
Two-plane (frontal)	3.13	0.4–4.8	3.11	0.51–4.52	0.379
Three-plane (total)	3.92	0.52–8.54	3.30	0.52–5.21	0.148

Median, range and non-parametric Wilcoxon *t*-test. *n* = 16

In seven slipped hips, the epiphysis moved in the cranial direction and nine in the caudal direction; on the non-slipped side, 15 moved cranially and one caudally. The median movement was 0.16 mm caudally (range 2.80 caudal to 3.58 cranial) on the slipped side and 2.28 mm cranial (range 0.02 caudal to 4.25 cranial) on the non-slipped side, *p* = 0.003 (Tables 2 and 3; Fig. 2b).

The epiphysis of two patients moved anteriorly on the slipped side, while in 14 patients, the movement was in a posterior direction. On the non-slipped side, three patients exhibited anterior movement and the remaining 13 posterior. The median movement in the anterior/posterior plane was posterior 2.28 mm (range 7.25 posterior to 1.33 anterior) on the slipped side and 0.91 mm posterior (range 2.95 posterior to 1.02 anterior) on the non-slipped side, *p* = 0.030 (Tables 2 and 3; Fig. 2c).

The vector of the movement in the frontal plane (medial/lateral and cranial/caudal) direction was on the slipped side median 3.13 mm (range 0.39–4.82 mm) and on the non-slipped side median 3.11 mm (range 0.51–4.52 mm), *p* = 0.379. The vector of the movement in the three planes together (total movement) was on the slipped side median 3.92 (range 0.52–8.54 mm) and on the non-slipped side 3.30 (range 0.52–5.21 mm), *p* = 0.148 (Table 3).

Although not significant, we found a tendency towards increased cranial movement in the younger patients and increased caudal movement in the older patients.

## Discussion

We have been able to measure movement of the epiphysis in relation to the metaphysis in the proximal femur after fixation of SCFE with a high level of precision. These movements indicate signs of continued growth after pinning in SCFE on both the slipped and prophylactically pinned non-slipped sides, with a marked increased growth on the non-slipped side.

One of the limitations of this study is the small number of participants, although, to our knowledge, this study includes the as-yet largest reported number. In addition, we only present a 1-year follow-up at this stage, and, hence, do not know how much more growth can be expected until skeletal maturity. A control group could not be used since this study involved surgery. The placement and the difficulty in spreading the tantalum markers through the narrow, drilled hole (6.5 mm in diameter) limited available measurements and the rotation of the epiphysis could not be assessed reliably and is, therefore, excluded. Furthermore, a vector along the length of the femoral neck could have facilitated the interpretation of the movements occurring. This was, however, not the primary aim of the study.

**Fig. 3:**
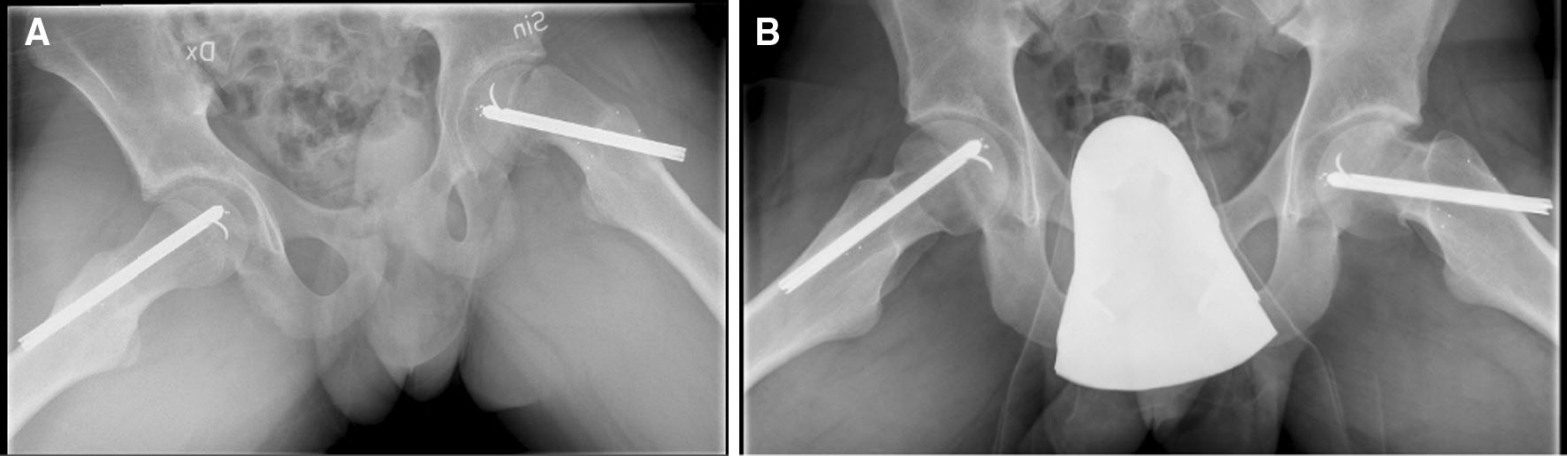
Radiograph immediately postoperative (**a**) and 3.4 years later (**b**), at skeletal maturity. Continued growth and remodelling can be detected, but it is difficult to define and quantify

Kumm et al. noted that the short thread screws in about one quarter of his patients needed to be replaced with a longer one due to growth [9]. Wong-Chung et al. used radiographs to assess remodelling and measured the degree of physeal shaft angle correction [15]. However, the accuracy of conventional radiograph measurements of continued growth after SCFE is difficult to assess [19, 23]. Kallio et al. used ultrasound and noted immediate remodelling of the epiphyseal and metaphyseal step within weeks of surgery [17]. Hägglund et al. used RSA to measure growth as the change of distance between individual tantalum markers placed on both sides of the physis and found growth of up to 15 mm until closure of the growth plate [11]. From our study, using several markers on both sides of the growth plate, we were able to quantify and perform a more complete analysis of the movements that occurred when compared with previous reports. We appreciate that further growth and, to some extent, remodelling can be detected on plain radiographs, but find it difficult to quantify (Fig. 3).

One would expect growth to be mainly cranial and medial on the slipped side, as was observed on the non-slipped, prophylactic pinned side in 15/16 patients. However, on the slipped side, only 8/16 showed cranial and medial movement. In the younger patients, there was a trend towards increased cranial growth on the slipped side, which would be expected. Although not significant, we speculate that, in the older patients showing more caudal movement, this represents instability after pinning and/or the absence of remaining growth. When assessing movement over time at 3, 6 and 12 months, we noted that one patient dropped substantially between 3 and 6 months, moving into the caudal direction. This normalised at 1 year when cranial movement occurred, and might imply that stable fixation was not achieved initially but that the patient recovered after some time and normal growth was resumed.

Posterior movement was noted during the first year in the majority of the patients on both the slipped and non-slipped sides. This could, to some extent, be an effect of the femoral neck not being perfectly aligned with the frontal plane at the reference (first) RSA examination. This could also possibly be an effect of normal bone development with decrease of femoral anteversion occurring with growth [24].

In many centres, the non-slipped side is treated with a prophylactic pin fixation at the same time as fixation is performed on the slipped side. We found a clear difference in movement between the slipped and non-slipped sides throughout the entire group of participants, although this could not be fully explored due to the lack of data on normal growth in this age group and at this location. Based on our data on the non-slipped side, we do, however, think that the influence of the pinning on growth was very limited, especially since the pattern of growth on the non-slipped side was very similar in all patients.

As to understanding normal expected growth, we are still referring to older datasets with less precise measurements [25]. There is still limited knowledge of normal expected growth and, therefore, it remains difficult to make comparisons.

A general interpretation of our data is that the proximal femoral physis grows with a very organised predictable pattern on the normal non-slipped side, but in a disorganised unpredictable pattern on the slipped side in SCFE. The variability of movement on the slipped side could be the net effect of many factors with variable influence, such as the degree of slip, the stability of the physeal region after fixation, the extent of any damage to the growth plate and the remaining growth potential (maturity). We speculate that, in some cases, the physis might need some time to recover after the slip and fixation, before growth recurs in a more normal direction. In case of movement in an opposite direction than expected which causes further deformity of the slip, the stability of the fixation is not adequate. In these cases, it would be of importance to limit weight-bearing after surgery. The non-slipped side, on the other hand, shows a good and homogenous movement pattern, indicating no major adverse effects on growth, as discussed above. For these reasons, we should learn more and possibly be more diligent following patients closer postoperatively, even if the non-slipped side is prophylactically pinned. Here, RSA could have a role, besides other possible other high-resolution/high-precision methods, to assess movement.

Femoral acetabulum impingement as a cause of hip pain and the development of early osteoarthritis after SCFE is frequently discussed [26]. Due to the increasing clinical problem, surgical procedures have evolved, including arthroscopy and open surgery with so-called ‘safe dislocation of the hip’; however, the indications for these procedures are, as yet, far from definitive [26, 27]. Dynamic fixation of SCFE by smooth pins or short threaded screws could lead to substantial growth and remodelling of the hip, preventing the development of impingement and reducing the need for these relatively extensive procedures with potentially serious complications.

## Conclusion

We were able to measure movement of the epiphysis in relation to the metaphysis after fixation with a smooth pin for slipped capital femoral epiphysis (SCFE), both on the slipped side and on the prophylactically fixed non-slipped side. This suggests that growth continues after using the smooth pin, especially on the non-slipped side. There was also an increased displacement of the epiphysis posteriorly on the slipped side, suggesting that minor instability remained after pinning in some patients.

The radiostereometric analysis (RSA) method can be used for further studies on the understanding of remodelling after ‘growth-sparing’ fixation of SCFE.
